# Psychiatric comorbidity: a concept in need of a theory

**DOI:** 10.1017/S0033291723001605

**Published:** 2023-10

**Authors:** Julie Nordgaard, Kasper Møller Nielsen, Andreas Rosén Rasmussen, Mads Gram Henriksen

**Affiliations:** 1 Mental Health Center Amager, University Hospital Copenhagen, Denmark; 2 Department of Clinical Medicine, University of Copenhagen, Denmark; 3 Center for Youth Mental Health, The University of Melbourne, Melbourne, Victoria, Australia; 4 Department of Communication, Center for Subjectivity Research, University of Copenhagen, Denmark

**Keywords:** differential diagnosis, hierarchy, reification, state, trait

## Abstract

Despite being a relatively new concept, psychiatric comorbidity, i.e. the co-occurrence of two or more mental disorders, has become widespread in clinical practice and psychiatric research. In this article, we trace the origin of the concept of psychiatric comorbidity, discuss the conceptual literature and point to basic problems concerning inadequate definition of the concept, differential diagnostic issues, and reification of mental disorders. We illustrate how these problems may have consequences for diagnostic assessment in current clinical practice and psychiatric research. To address some of the problems related to psychiatric comorbidity, we discuss potential principles for assessing psychiatric comorbidity. Inspired by Feinstein's original concept of comorbidity in general medicine and his differential diagnostic principles, we emphasize the importance of *independence* of mental disorders when assessing psychiatric comorbidity. We suggest that knowledge of *trait v*. *state* conditions and of the multitudinous clinical manifestations beyond what is captured in the diagnostic manuals may be helpful for assessing the independence of mental disorders and thus psychiatric comorbidity. We further argue that a more hierarchical diagnostic system and explicit exclusionary rules could improve clinical practice and research by reducing informational complexity and combating unwarranted psychiatric comorbidity.

## Introduction

Despite being a relatively new concept, comorbidity has gained immense popularity in psychiatry and is currently widespread in everyday clinical work and research. The concept of comorbidity was introduced to general medicine in 1970 by Feinstein (1970), who defined it as ‘any distinct additional clinical entity that has existed or that may occur during the clinical course of a patient who has the index disease under study’. The concept of comorbidity was first used in psychiatry during the 1980s, and comorbidity was defined as the co-occurrence of medical conditions and/or mental disorders (Boyd et al., 1984; Klerman, 1990; Leckman, Weissman, Merikangas, Pauls, & Prusoff, 1983). Comorbidity was also used ‘internally’ in psychiatry to diagnose the co-occurrence of two or more mental disorders, viz. psychiatric comorbidity (Maj, 2005*b*).

The number of patients diagnosed with psychiatric comorbidity has increased significantly and empirical research on comorbid mental disorders has similarly grown (Lilienfeld, Waldman, & Israel, 1994; Lilienfeld et al., 2015). This development is also reflected in the diagnostic manuals, where the term ‘comorbid’ appeared 0 times in DSM-III but more than 600 times in DSM-5 (American Psychiatric Association, 2013). Despite the popularity of the concept of psychiatric comorbidity, it has often been criticized. In fact, there is a striking incongruence between the widespread use of psychiatric comorbidity in clinical practice and empirical research (e.g. Etchecopar-Etchart et al., 2021; Plana-Ripoll et al., 2019; Strålin & Hetta, 2019) and theoretical studies that generally pose strong reservations toward psychiatric comorbidity (e.g. First, 2005; Frances, Widiger, & Fyer, 1990; Lilienfeld et al. 1994; Maj, 2005*b*; Meehl, 2001; van Praag, 1996; Vella, Aragona, & Alliani, 2000). As Maj has emphasized, this persistent incongruence calls for critical reflection on the concept of psychiatric comorbidity (Maj, 2005*a*).

The purpose of this study is to examine the concept of psychiatric comorbidity and its impact on everyday clinical work and psychiatric research. First, we trace the origin of the concept of psychiatric comorbidity back to Feinstein's concept of comorbidity, and discuss some basic problems related to psychiatric comorbidity. We then exemplify how such problems may affect research and clinical practice. Finally, inspired by Feinstein's definition of comorbidity and his differential diagnostic principles, we discuss principles for assessing psychiatric comorbidity.

## Theoretical issues

Feinstein (1970, p. 457) offered several examples to illustrate his concept of distinct additional clinical entity – e.g. ‘If the index disease is primary cancer of the lung, the co-morbid diseases can be such entities as: episodes of mumps or bleeding duodenal ulcer that occurred antecedently; coronary artery disease or pneumococcal pneumonia detected when the cancer is discovered’. In brief, Feinstein's (1970; Kaplan & Feinstein, 1974) discussion of comorbidity gravitates around the case of cancer with a pragmatic focus on identifying comorbid diseases or conditions that may affect diagnosis, prognosis, pathogenicity, or therapeutic outcome. Since the concept of comorbidity invariably raises differential diagnostic issues, Feinstein proposed differential diagnostic principles for assessing comorbidity, emphasizing *toponymy* (i.e. considering an event's anatomic location in relation to that of the index disease) and *chronometric reasoning* (i.e. events or conditions, prior or presently, that impact the natural course of the index disease). From Feinstein's original definition, the many examples he discussed in the article, and the character of the differential diagnostic principles, it is evident that his concept of comorbidity hinges on the notion of *distinct additional clinical entities*, and, most importantly, that to qualify as a *distinct additional clinical entity* a medical condition must have either *known etiology* and/or *circumscribed pathology* (Lilienfeld et al., 1994; Maser & Cloninger, 1990; Meehl, 2001; Vella et al., 2000; Wyrsch, 1930).

In the 1980s, Feinstein's concept of comorbidity found its way into psychiatry, where it was also used to diagnose psychiatric comorbidity, i.e. the *co-occurrence of two or more mental disorders* (Andrews, Henderson, & Hall, 2001; Klerman, 1990; Maser & Cloninger, 1990; Plana-Ripoll et al., 2019; Trull, Scheiderer, & Tomko, 2012; Wittchen, 1996). While this may seem like an intuitive extension of Feinstein's definition, it is, in fact, not unproblematic.

When it comes to psychiatric comorbidity, it is, in most cases, not clear that the founding principle of Feinstein's original definition, viz., the notion of *distinct additional clinical entities*, can be met. To qualify as distinct additional clinical entities, mental disorders must have either known etiology or circumscribed pathology. Most mental disorders, however, have unknown etiology. Psychiatric comorbidity is therefore typically based solely upon description of psychopathology. If mental disorders had well circumscribed psychopathology, it could potentially compensate for the absence of known etiology, and they would still qualify as distinct additional clinical entities. However, mental disorders are precisely *not* assumed to be completely demarcated from each other in the sense that single symptoms of one mental disorder cannot occur in another disorder – e.g. as stated in DSM-5-TR (American Psychiatric Association, 2022; cf. American Psychiatric Association, 1980, p. 6), ‘there is no assumption that each category of mental disorder is a completely discrete entity with absolute boundaries dividing it from other mental disorders or from no mental disorder’. This assumption, underlying the classification of mental disorders, is natural given that many symptoms are found across mental disorders (e.g. anxiety), and only few symptoms are considered diagnostically specific for any disorder (e.g. Mølstrøm, Henriksen, & Nordgaard, 2020). However, the absence of known etiology and circumscribed psychopathology jointly implies that the founding principle of Feinstein's concept of comorbidity, i.e. the notion of distinct additional clinical entities, typically is lacking in psychiatry.

Recognizing this, First (2005) proposed a distinction between three types of psychiatric comorbidity: (1) *true comorbidity* pertaining to cases of mental disorders that fulfil Feinstein's requirement of distinct additional clinical entities; (2) *artifactual comorbidity* which is a by-product of the diagnostic manuals’ decision ‘to “split” diagnostic entities into numerous specific narrowly-defined disorders’ (First, 2005, p. 207); and (3) *spurious comorbidity* which is best avoided (e.g. comorbidity between autistic disorder and Asperger's disorder in DSM-IV). Since *true comorbidity* is rare in psychiatry and *spurious comorbidity* must be avoided, psychiatric comorbidity, First (2005) argued, primarily consists of cases of *artifactual comorbidity*. Consequently, First (2005, p. 206) noted that it could be misleading to speak of psychiatric comorbidity – ‘It is important to understand that comorbidity in psychiatry does not imply the presence of multiple diseases or dysfunctions’. Still, First advocated the use of psychiatric comorbidity, citing the instruction from ICD-10 (World Health Organization, 1992), recommending that ‘clinicians should follow the general rule of recording as many diagnoses as are necessary to cover the clinical picture’. However, in the context of ICD-10, this instruction does not imply that a diagnosis should be made for every symptom constellation (mental disorder) for which a patient fulfils diagnostic criteria. In ICD-10, it only pertains to symptom complexes that cannot be accommodated by the index disorder. For example, a diagnosis of schizophrenia is sufficient to cover almost all other symptoms – e.g. anxiety and changes of personality are common features of schizophrenia. Since such symptoms can be accommodated by the index disorder (schizophrenia), no additional diagnosis is typically needed ‘to cover the clinical picture’. Finally, the renouncement of the founding principle of comorbidity (i.e. distinct additional clinical entities) seems premature and leaves us with a concept of psychiatric comorbidity (artifactual comorbidity) that, strictly put, negates the very meaning of the concept of comorbidity. If the concept of psychiatric comorbidity is to be meaningful, a qualification of distinct additional clinical entities other than known etiology and circumscribed pathology is needed (see the final section for a proposal).

Another issue, complicating matters further, is the fact that the diagnostic criteria, as stated in ICD-10 (World Health Organization, 1992), ‘do not pretend to be comprehensive statements about the current state of knowledge of the disorders’ but serve only as ‘a reasonable basis for defining the limits of categories’. In other words, the purpose of the diagnostic criteria is to *delimit* the different mental disorders from each other; it is not to exhaustively describe their psychopathology. Although the diagnostic criteria initially were considered as ‘gate keepers’, i.e. a sort of minimum requirement for making a diagnosis, they have increasingly come to be perceived as almost exhaustive of the psychopathology of the different mental disorders (Andreasen, 2007; Kendler, 2016*a*; Kendler, 2022; Kendler & Zachar, 2008). This perception is, to some extent, underpinned by contemporary textbooks of psychiatry that regularly are tailored to the diagnostic criteria and therefore predominately include information about psychopathology listed as diagnostic criteria. Consequently, knowledge of psychopathology that is not included as diagnostic criteria is disappearing from contemporary psychiatric discourse and research (Parnas, 2011). This unintended development may also impact assessment of comorbidity. For example, clinicians or researchers may be unaware that many symptoms and signs are well-known features of a given disorder (e.g. schizophrenia) when such symptoms and signs (e.g. obsessive-like features, concentration and attentional difficulties, self-disorders, etc.; Henriksen, Raballo, & Nordgaard, 2021; Rasmussen & Parnas, 2022) are not included in the diagnostic criteria for the disorder.

The final theoretical issue that we will address here can be summarized under the heading of reification, which designates that something essentially non-thing-like acquires a thing-like status. As Kendler (2016*b*) aptly put it, ‘It is a conceptual error to assume that our diagnostic criteria constitute rather than index our disorders’. Despite such cautionary statements, the diagnostic categories are often taken to be the mental disorders they index (Frances et al., 1990; Hyman, 2010; Jablensky, 2004, 2005; Kendler, 2014). Already in the 1970s, Kendell (1975) eloquently pointed to this crucial issue, ‘Because the categories and those attributed to it has a name, it acquires a shadowy ‘existence’ of its own, and it eventually comes to be assumed that its members must differ in some fundamental way from members of the other categories in the typology’. Reification is important for the discussion of psychiatric comorbidity because reification of mental disorders and psychiatric comorbidity to some extent seem to implicate or reinforce each other. Even though the diagnostic manuals repeatedly emphasize the absence of any assumption of clear-cut boundaries between the diagnostic categories, the categories nonetheless, as Kendell described, tend to reify over time (Hyman, 2010; Kendell & Jablensky, 2003) and come to be viewed as separate entities.

Although psychiatric comorbidity in the literature usually is defined as the co-occurrence of mental disorders, i.e. without *explicitly* assuming their independence (for an exception, see Caron & Rutter, 1991), the concept of psychiatric comorbidity nonetheless carries an *implicit* assumption of *independence* of mental disorders. This implicit assumption of independence of mental disorders permeates much research on psychiatric comorbidity. For example, a large-scale Danish register study found that all mental disorders were associated with an increased risk of all other mental disorders (Plana-Ripoll et al., 2019). This finding must, however, be tempered by the fact that the hierarchical rules of the applied diagnostic manual (ICD-10), which exert rules for diagnosing comorbidity, were not applied in the study. Another study – the Dunedin Longitudinal Study from New Zealand – also found psychiatric comorbidity to a large extent on a lifetime basis in a complete birth cohort (Caspi & Moffitt, 2018). Here, psychiatric comorbidity was assessed ‘without regard for hierarchical exclusionary rules’ (Caspi et al., 2014). In both studies, mutual independence of all mental disorders was presumed, exemplifying to some extent a reified approach to mental disorders, and the studies’ findings of widespread psychiatric comorbidity rest in the end on an inadequate application of the guidelines of the relevant diagnostic manual. This prompts the question of whether such results are perhaps less facts than artifacts, resulting from insufficient differential diagnosis?

## Clinical issues

The theoretical issues discussed above also seep into daily clinical reality, which we illustrate with two clinical vignettes.

### Clinical vignette 1

A 28-year-old man was diagnosed with schizophrenia when he was 22. He had had a difficult childhood with mentally ill parents. In primary school, he had some difficulties concentrating and motor restlessness. He only had a few friends. He dropped out of high school because of severe concentration problems. He started different educations and jobs, but he was unable to maintain them for more than a few months. In the last couple of years, he has not been working or getting an education.

He was diagnosed with schizophrenia due to the presence of delusions, auditory hallucinations, negative symptoms, and social difficulties. He was in treatment with antipsychotic medication for several periods with some effect. At age 28, he was diagnosed with comorbid attention-deficit/hyperactivity disorder (ADHD) due to concentration problems, difficulties in completing simple assignments, and motor restlessness. It was assessed that these difficulties had been present since childhood due to his concentration difficulties and motor restlessness in primary school.

The challenge in this case is that some symptoms of ADHD resemble some symptoms of schizophrenia – motor disturbances, concentration difficulties, and cognitive impairments are well-known features of schizophrenia (Elvevåg & Goldberg, 2000). Consequently, it is difficult to determine if these symptoms are genuine symptoms of ADHD or simply early developmental aspects of schizophrenia. However, deciding whether these symptoms are expressive of ADHD or schizophrenia can have implications for treatment. If these symptoms are considered reflective of comorbid ADHD, this can lead to prescription of CNS stimulants such as methylphenidate, which some studies have reported may lead to psychotic activation in patients with schizophrenia (Lieberman, Kane, & Alvir, 1987).

### Clinical vignette 2

A 28-year-old man, who recently had been diagnosed with obsessive–compulsive disorder (OCD), was subsequently diagnosed with schizophrenia due to the presence of persecutory and self-referential delusions, visual hallucinations, and negative symptoms. He described obsessions and compulsions with several themes, especially washing compulsions related to intrusive mental images of contamination and infection. When cooking, he watches ‘whole movies in the head’ about this issue. The involuntary images are extremely vivid, last several minutes, and ‘kind of automatically zoom in on disgusting details’, e.g. that some meat accidentally touched the cutlery, or that some animal urinated on the vegetables. He claims to be aware that these thoughts are exaggerated, but they still make him anxious. Often, there are several, unrelated scenarios occurring at the same time, which he ‘observes in the head’; ‘it is like a high-way intersection’, he states, the ‘thoughts are like cars passing in all directions’. When such ‘OCD-thoughts’ become intense, they feel alien, ‘almost as-if they were not mine’. He is occupied with obsessions and compulsion for several hours daily and avoids eating outside his home.

Here, OCD symptomatology is present in a patient, who also fulfils the diagnostic criteria for schizophrenia. In DSM-5 (American Psychiatric Association, 2013, p. 237), criterion D specifies that a diagnosis of OCD should not be made if the disturbance is better explained by another diagnosis. It is certainly possible that the OCD symptomology in this patient is a part of schizophrenia. Yet, DSM-5 also states, ‘OCD is also much more common in individuals with certain other disorders (…) when one of those other disorders is diagnosed, the individual should be assessed for OCD as well. For example, in individuals with schizophrenia or schizoaffective disorder, the prevalence of OCD is approximately 12%’ (American Psychiatric Association, 2013, p. 242). How is the clinician to know whether the obsessions and compulsions are ‘better explained’ by schizophrenia (which would be the case in ICD-10 due to its hierarchical principles) or if making a comorbid diagnosis of OCD is the sound clinical judgment? Although the patient demonstrates some degree of insight, he is unable to resist or suppress the intrusive thoughts and images, which occur automatically with a weakened sense of being generated by the patient. In classical psychopathological literature, such symptoms exemplify pseudo-obsessive phenomena, which are characteristic of schizophrenia spectrum disorders (for reviews, see Bürgy, 2007; Rasmussen & Parnas, 2022).

As illustrated in the vignettes, applying the concept of psychiatric comorbidity in clinical settings faces certain problems. In medicine, it is an honored principle to prefer to allocate a single diagnosis if that diagnosis can accommodate all the patient's pathological phenomena. In both vignettes, the patients’ symptomatology can be explained by a single diagnosis (schizophrenia), but it is not entirely clear whether a comorbid diagnosis should be made. Following Feinstein (1970), diagnostically unspecific symptoms, which can be seen across a range of disorders, should not lead to a comorbid diagnosis, if they possibly are a part of the index disease. Nonetheless, this type of psychiatric comorbidity – what First called *artifactual comorbidity* – appears to be widespread in clinical work and research, but here it is often uninformed by First's realization that much psychiatric comorbidity does not qualify as *true comorbidity*. If the hierarchical rules of the diagnostic manuals are ignored or their exclusionary rules are too vague, it carries a risk of inflating comorbidity by reducing the psychopathological complexity of mental disorders to mere separate ‘symptom-compartments’. In clinical practice, it additionally carries a risk of ‘compartmentalizing’ the psyche of patient, thereby not seeing the patient as a whole and unified human being but instead as a person with a psyche composed of, say, one part schizophrenia, one part OCD, and one part ADHD, and where each of these parts may invite different disorder-specific treatments. Receiving comorbid diagnoses may also lead to unwarranted polypharmacy (Maj, 2005*b*) and obstruct other kinds of treatment.

Diagnosing psychiatric comorbidity also entails ethical issues. If the diagnostic guidelines are not adequately followed, we are dealing with cases of misdiagnosis, which is both unethical and harmful. A related question is if a patient's symptomology can be explained by a single diagnosis, is it then ethically sound to diagnose the patient with multiple mental disorders? If we make multiple diagnoses to explain the same amount of psychopathology that could be explained by a single diagnosis, do we not risk putting the patient in an even more difficult situation? Receiving one diagnosis is frequently experienced as difficult, often requiring adjustment of one's self-understanding to accommodate certain disorder-specific vulnerabilities and developing suitable protective or compensatory strategies to deal with them. This difficulty likely increases with the number of received diagnoses and vulnerabilities one faces.

Finally, the current use of psychiatric comorbidity also has implications for research. For example, in the case of comorbid mental disorders, which disorder is considered the index disorder in register or empirical studies? If a disorder placed lower in the diagnostic hierarchy (ICD-10) is considered the index disorder and a higher placed comorbid disorder is ignored, then symptoms traditionally restricted to the higher placed disorder (e.g. psychosis) may also be found in the lower placed disorder. Regularly, such transdiagnostic findings are used to support the claim that a dimensional approach to the classification of mental disorders fits the data better than a categorical approach (Clark, 2005; Cohen & Öngür, 2023). Yet, we wonder if these data fit a dimensional approach better because it was collected with a liberal assessment of psychiatric comorbidity, disregarding hierarchical rules and/or exclusion criteria? Finally, we wonder if, say, OCD in a patient with only OCD is really the same as OCD in a patient with comorbid schizophrenia? Do we not risk losing sight of essential qualitative differences between mental disorders if we disregard the overall psychopathological context (Gestalt) in which the symptoms occur and from which they receive their clinical significance?

## Toward a new assessment of psychiatric comorbidity

Despite the reservations outlined above, we believe the concept of psychiatric comorbidity can be both appropriate and helpful in psychiatry. There are clinical situations, where it is relevant to make a comorbid diagnosis – e.g. a patient, who was diagnosed with a personality disorder 20 years ago, and who develops a major depression. In this case, the patient's mental condition, which has been stable for years, changes markedly with new and clearly depressive symptoms emerging. However, if the concept of psychiatric comorbidity is to be applied in a more meaningful way in psychiatry, its theoretical foundation must first be established. Inspired by Feinstein's concept of comorbidity and his differential diagnostic principles, we suggest some potential principles for an improved assessment of psychiatric comorbidity.

Since known etiology and circumscribed pathology cannot adequately qualify mental disorders as distinct additional clinical entities, we must look elsewhere for a principle to secure, as far as possible, their *mutual independence*. Here, it may be helpful to distinguish between two different levels of analysis: a diagnostic level and a nosological level.

**Figure. 1.**
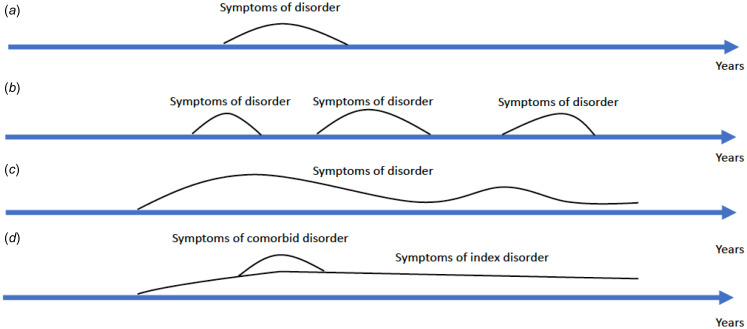
Illustration of state and trait conditions. (*a*) A state condition with a single episode of a disorder, e.g. major depression. (*b*) A state condition with recurring episodes, e.g. bipolar disorder. (*c*) A trait condition, e.g. schizophrenia. (*d*) A trait condition with a comorbid state condition, e.g. personality disorder with a single episode of major depression.

The diagnostic level targets assessment of comorbidity in clinical practice and research using the existing diagnostic manuals. Here, we suggest that the distinction between *trait* and *state* conditions (see Fig. 1) can play a more crucial role in determining whether a patient's symptomatology amounts to a distinct clinical entity in addition to the index disorder. *Trait conditions* are mental disorders, which are defined as such in the diagnostic manuals. The symptomatology of trait conditions can fluctuate in severity, but the disorder is usually present for a longer time, often years. Examples of trait conditions can be autism spectrum disorder, ADHD, schizophrenia, or personality disorders. By contrast, state conditions are mental disorders, which are defined as such in diagnostic manuals, and which are present in one or more circumscribed episodes. Examples of state conditions can be affective disorders, e.g. major depression. In the example above, the patient has a stable trait condition (a personality disorder), when a later state condition (major depression) emerges. Crucially, the patient's more enduring symptomology is accommodated by the personality disorder, and the emerging depressive symptoms, which cannot be explained by the trait condition, are accommodated by the diagnosis of major depression. If, by contrast, we met the patient for the very first time, and the patient fulfilled criteria for both major depression and a personality disorder, we should not immediately make both diagnoses, since apparent changes of personality could be a product of the current depressive episode. In this case, we should diagnose and treat the major depression, and wait until the depression recedes to see if the changes of personality recede with it. If they do not recede, we could then perhaps diagnose a comorbid personality disorder.

Applying the distinction between trait and state conditions to inform assessment of psychiatric comorbidity cannot, however, stand alone. Such an assessment ultimately rests on a comprehensive understanding of the psychopathology of mental disorders. Such an understanding, which necessarily goes beyond the limited range of symptoms and signs included in the major diagnostic manuals, forms an indispensable part of the ‘clinical judgment’ upon which diagnoses must be made (American Psychiatric Association, 2022). Such an understanding can only be achieved through varied clinical experience, reading psychopathological literature, and ongoing discussions about psychopathological phenomena with experts.

Assessment of comorbidity can be substantially improved at the diagnostic level by making suitable changes at the nosological level. Here, a classical principle for differential diagnosis can further help us restrict the use of psychiatric comorbidity. Traditionally, psychiatry has operated with a hierarchical classification of mental disorders (American Psychiatric Association, 1980), based on the clinical method (Owen, 2023), which, in a descending order, roughly can be summarized as follows: organic disorders, schizophrenia, affective disorders, anxiety disorders, and personality disorders (Jaspers, 1997, pp. 605–606, 611–612; Klerman, 1990; Owen, 2023, p. 1704). For disorders at the top of the diagnostic hierarchy (e.g. organic disorders or schizophrenia), most, if not all, symptoms may occur. By contrast, the range of symptoms is much more limited in disorders placed lower in the diagnostic hierarchy (e.g. anxiety or personality disorders) (American Psychiatric Association, 1980). Thus, if a patient fulfils diagnostic criteria for more than one disorder, the highest placed disorder generally outranks lower placed disorders, implying that comorbid diagnoses should not be made if the symptomatology can be accommodated by the index disorder – a point vividly illustrated in the decision trees for differential diagnoses in previous editions of DSM (American Psychiatric Association, 1980, pp. 339–349, 2000, pp. 744–757) and *still* operative in ICD-10.

Today, emphasis on the diagnostic hierarchy of mental disorders has gradually eroded, as it has been argued that the hierarchy is empirically unfounded (e.g. Boyd et al., 1984; Leckman et al., 1983; Pincus, Tew, & First, 2004). This crumbling of a pillar of classical medical thinking, coupled with a somewhat blind faith in empiricism (Faust & Miner, 1986; Kendler, 1990), has, however, had various ‘unintended consequences’ (Andreasen, 2007; Wakefield, 2016). In the case of psychiatric comorbidity, the weakening of the diagnostic hierarchy may have contributed to the inflation of psychiatric comorbidity, because symptom constellations that previously would have been regarded as mere psychopathological *aspects* of hierarchically higher placed disorders – e.g. anxiety or changes of personality as features of schizophrenia or major depression – now often are perceived as comorbidities. Despite going against current trends, opting for a more hierarchical diagnostic system, which is both simple to understand and easy to administer, could improve clinical practice and psychiatric research by reducing informational complexity and combating ‘compartmentalization’ of the human psyche and a superficial and partial understanding of patients’ difficulties (Aragona, 2009; Jablensky, 2004; Widiger & Ford-Black, 1994).

Two often invoked arguments against a diagnostic hierarchy are that it prevents a full representation of patients’ symptomatology and that it restricts research into lower placed disorders (Clark, 2005). The first objection hinges on the erroneous assumption that the diagnostic criteria are exhaustive of the psychopathology of the different disorders. Regarding the second objection, we cannot see why research on lower placed disorders necessarily would be restricted. Moreover, it could be beneficial to study such disorders when they occur without comorbidity – is anxiety (occurring without a comorbid disorder) really the same condition as anxiety occurring in, e.g. schizophrenia?

Even without a strict hierarchical diagnostic system, psychiatric comorbidity may still be restricted using exclusion criteria (Robins & Guze, 1970). While exclusion criteria are frequently used, they have become increasingly vague and open to interpretation, exemplified by the recurring exclusion criterion in DSM-5 stating that the disturbance is ‘not better explained’ by another disorder. How can we decide if symptoms of a given disorder is ‘better explained’ by another disorder and what is the basis of that judgment? More explicit exclusion criteria (e.g. diagnosis A must not be made if diagnosis B or C is made) may diminish the role of interpretation and restrict psychiatric comorbidity.

Finally, other criteria for diagnostic validity have been proposed (e.g. biological markers, treatment response, or risk factors) but none of them seem able to provide strong differential diagnostic tools as long as the etiology and pathogenesis of most mental disorders remain largely unknown (e.g. Robins & Guze, 1970; and the validators for change of diagnostic criteria at the DSM proposal submission portal).

## Conclusion

We addressed theoretical issues related to the concept of psychiatric comorbidity and illustrated how these issues may affect clinical practice and psychiatric research. If the concept of psychiatric comorbidity is to be applied in a scientific sense, it is important to clarify in what sense mental disorders can be said to be independent from each other and thus constitute distinct additional clinical entities. At a diagnostic level, we suggested that the distinction between trait and state conditions may be helpful in combination with comprehensive psychopathological knowledge. At a nosological level, we suggested that a more hierarchical diagnostic system and/or more explicit exclusionary rules may aid differential diagnosis and pave the path to a sounder application of psychiatric comorbidity with benefits for both clinical practice and research.

## References

[ref1] American Psychiatric Association (1980). Diagnostic and statistical manual of mental disorders (3rd ed.). Arlington: American Psychiatric Association.

[ref2] American Psychiatric Association (2000). Diagnostic and statistical manual of mental disorders (4th ed., text revised). Arlington: American Psychiatric Association.

[ref3] American Psychiatric Association (2013). Diagnostic and statistical manual of mental disorders (5th ed.). Arlington: American Psychiatric Association.

[ref4] American Psychiatric Association (2022). Diagnostic and statistical manual of mental disorders (5th ed., text revised). Arlington: American Psychiatric Association.

[ref5] Andreasen, N. C. (2007). DSM and the death of phenomenology in America: An example of unintended consequences. Schizophrenia Bulletin, 33, 108–112. 17158191 10.1093/schbul/sbl054 PMC2632284

[ref6] Andrews, G., Henderson, S., & Hall, W. (2001). Prevalence, comorbidity, disability and service utilisation. Overview of the Australian National Mental Health Survey. British Journal of Psychiatry, 178, 145–153. 10.1192/bjp.178.2.145 11157427

[ref7] Aragona, M. (2009). The role of comorbidity in the crisis of the current psychiatric classification system. Philosophy, Psychiatry, & Psychology, 16, 1–11.

[ref8] Boyd, J. H., Burke, J. D. Jr., Gruenberg, E., Holzer, C. E. 3rd, Rae, D. S., George, L. K., … Nestadt, G. (1984). Exclusion criteria of DSM-III. A study of co-occurrence of hierarchy-free syndromes. Archives of General Psychiatry, 41, 983–989. 6477056 10.1001/archpsyc.1984.01790210065008

[ref9] Bürgy, M. (2007). Obsession in the strict sense: A helpful psychopathological phenomenon in the differential diagnosis between obsessive-compulsive disorder and schizophrenia. Psychopathology, 40, 102–110. 17215596 10.1159/000098490

[ref10] Caron, C., & Rutter, M. (1991). Comorbidity in child psychopathology: Concepts, issues and research strategies. Journal of Child Psychology and Psychiatry and Allied Disciplines, 32, 1063–1080. 1787137 10.1111/j.1469-7610.1991.tb00350.x

[ref11] Caspi, A., Houts, R. M., Belsky, D. W., Goldman-Mellor, S. J., Harrington, H., Israel, S., … Moffitt, T. E. (2014). The *p* factor: One general psychopathology factor in the structure of psychiatric disorders? Clinical Psychological Science, 2, 119–137. 25360393 10.1177/2167702613497473 PMC4209412

[ref12] Caspi, A., & Moffitt, T. E. (2018). All for one and one for all: Mental disorders in one dimension. American Journal of Psychiatry, 175, 831–844. 29621902 10.1176/appi.ajp.2018.17121383 PMC6120790

[ref13] Clark, L. A. (2005). Temperament as a unifying basis for personality and psychopathology. Journal of Abnormal Psychology, 114, 505–521. 16351374 10.1037/0021-843X.114.4.505

[ref14] Cohen, B. M., & Öngür, D. (2023). The need for evidence-based updating of ICD and DSM models of psychotic and mood disorders. Molecular Psychiatry. 10.1038/s41380-023-01967-7 36697753

[ref15] Elvevåg, B., & Goldberg, T. E. (2000). Cognitive impairment in schizophrenia is the core of the disorder. Critical Reviews in Neurobiology, 14, 1–21. 11253953

[ref16] Etchecopar-Etchart, D., Korchia, T., Loundou, A., Llorca, P. M., Auquier, P., Lançon, C., … Fond, G. (2021). Comorbid major depressive disorder in schizophrenia: A systematic review and meta-analysis. Schizophrenia Bulletin, 47, 298–308. 33252130 10.1093/schbul/sbaa153 PMC8451068

[ref17] Faust, D., & Miner, R. A. (1986). The empiricist and his new clothes: DSM-III in perspective. American Journal of Psychiatry, 143, 962–967. 3728742 10.1176/ajp.143.8.962

[ref18] Feinstein, A. R. (1970). The pre-therapeutic classification of co-morbidity in chronic disease. Journal of Chronic Diseases, 23, 455–468. 26309916 10.1016/0021-9681(70)90054-8

[ref19] First, M. B. (2005). Mutually exclusive versus co-occurring diagnostic categories: The challenge of diagnostic comorbidity. Psychopathology, 38, 206–210. 16145276 10.1159/000086093

[ref20] Frances, A. J., Widiger, T., & Fyer, M. R. (1990). The influence of classification methods on comorbidity. In J. D. Maser & C. R. Cloninger (Eds.), Comorbidity of mood and anxiety disorders (pp. 41–59). Washington: American Psychiatric Press, Inc.

[ref21] Henriksen, M. G., Raballo, A., & Nordgaard, J. (2021). Self-disorders and psychopathology: A systematic review. The Lancet Psychiatry, 8, 1001–1012. 34688345 10.1016/S2215-0366(21)00097-3

[ref22] Hyman, S. E. (2010). The diagnosis of mental disorders: The problem of reification. Annual Review of Clinical Psychology, 6, 155–179. 10.1146/annurev.clinpsy.3.022806.091532 17716032

[ref23] Jablensky, A. (2004). The syndrome – an antidote to spurious comorbidity? World Psychiatry, 3, 24–25. PMC1414655 16633445

[ref24] Jablensky, A. (2005). Categories, dimensions and prototypes: Critical issues for psychiatric classification. Psychopathology, 38, 201–205. 16145275 10.1159/000086092

[ref25] Jaspers, K. (1997). General psychopathology. London: John Hopkins University Press.

[ref26] Kaplan, M. H., & Feinstein, A. R. (1974). The importance of classifying initial co-morbidity in evaluating the outcome of diabetes mellitus. Journal of Chronic Diseases, 27, 387–404. 4436428 10.1016/0021-9681(74)90017-4

[ref27] Kendell, R., & Jablensky, A. (2003). Distinguishing between the validity and utility of psychiatric diagnoses. American Journal of Psychiatry, 160, 4–12. 12505793 10.1176/appi.ajp.160.1.4

[ref28] Kendell, R. E. (1975). The role of diagnosis in psychiatry. Oxford: Blackwell Scientific Publications.

[ref29] Kendler, K. S. (1990). Toward a scientific psychiatric nosology. Strengths and limitations. Archives of General Psychiatry, 47, 969–973. 2222134 10.1001/archpsyc.1990.01810220085011

[ref30] Kendler, K. S. (2014). DSM issues: Incorporation of biological tests, avoidance of reification, and an approach to the ‘box canyon problem’. American Journal of Psychiatry, 171, 1248–1250. 25756765 10.1176/appi.ajp.2014.14081018

[ref31] Kendler, K. S. (2016 *a*). The phenomenology of major depression and the representativeness and nature of DSM criteria. American Journal of Psychiatry, 173, 771–780. 27138588 10.1176/appi.ajp.2016.15121509

[ref32] Kendler, K. S. (2016 *b*). Phenomenology of schizophrenia and the representativeness of modern diagnostic criteria. JAMA Psychiatry, 73, 1082–1092. 27626788 10.1001/jamapsychiatry.2016.1976

[ref33] Kendler, K. S. (2022). Potential lessons for DSM from contemporary philosophy of science. JAMA Psychiatry, 79, 99–100. 34878514 10.1001/jamapsychiatry.2021.3559

[ref34] Kendler, K. S., & Zachar, P. (2008). The incredible insecurity of psychiatric nosology. In K. S. Kendler & J. Parnas (Eds.), Philosophical issues in psychiatry: Explanation, phenomenology, and nosology (pp. 368–383). Baltimore: Johns Hopkins University Press.

[ref35] Klerman, G. L. (1990). Approaches to the phenomena of comorbidity. In Maser, J. D., & Cloninger, C. R. (Eds.), Comorbidity of mood and anxiety disorders (pp. 13–37). Washington: American Psychiatric Press, Inc.

[ref36] Leckman, J. F., Weissman, M. M., Merikangas, K. R., Pauls, D. L., & Prusoff, B. A. (1983). Panic disorder and major depression. Increased risk of depression, alcoholism, panic, and phobic disorders in families of depressed probands with panic disorder. Archives of General Psychiatry, 40, 1055–1060. 6625853 10.1001/archpsyc.1983.01790090017002

[ref37] Lieberman, J. A., Kane, J. M., & Alvir, J. (1987). Provocative tests with psychostimulant drugs in schizophrenia. Psychopharmacology, 91, 415–433. 2884687 10.1007/BF00216006

[ref38] Lilienfeld, S. O., Sauvigné, K. C., Lynn, S. J., Cautin, R. L., Latzman, R. D., & Waldman, I. D. (2015). Fifty psychological and psychiatric terms to avoid: A list of inaccurate, misleading, misused, ambiguous, and logically confused words and phrases. Frontiers in Psychology, 6, 1100. 26284019 10.3389/fpsyg.2015.01100 PMC4522609

[ref39] Lilienfeld, S. O., Waldman, I. D., & Israel, A. C. (1994). A critical examination of the use of the term and concept of *comorbidity* in psychopathology research. Clinical Psychology: Science and Practice, 1, 71–83.

[ref40] Maj, M. (2005 *a*). The aftermath of the concept of ‘psychiatric comorbidity’. Psychotherapy and Psychosomatics, 74, 67–68. 15741755 10.1159/000083164

[ref41] Maj, M. (2005 *b*). ‘Psychiatric comorbidity’: An artefact of current diagnostic systems? British Journal of Psychiatry, 186, 182–184. 10.1192/bjp.186.3.182 15738496

[ref42] Maser, J. D., & Cloninger, C. R. (1990). Comorbidity of anxiety and mood disorders: Introduction and overview. In Maser, J. D., & Cloninger, C. R. (Eds.), Comorbidity of mood and anxiety disorders (pp. 3–12). Washington: American Psychiatric Press, Inc.

[ref43] Meehl, P. E. (2001). Comorbidity and taxometrics. Clinical Psychology: Science and Practice, 8, 507–519.

[ref44] Mølstrøm, I.-M., Henriksen, M. G., & Nordgaard, J. (2020). Differential-diagnostic confusion and non-specificity of affective symptoms and anxiety: An empirical study of first-admission patients. Psychiatry Research, 291, 113302. 32763555 10.1016/j.psychres.2020.113302

[ref45] Owen, G. (2023). What is formulation in psychiatry? Psychological Medicine, 53, 1–8. 10.1017/S0033291723000016 PMC10106283 36878884

[ref46] Parnas, J. (2011). A disappearing heritage: The clinical core of schizophrenia. Schizophrenia Bulletin, 37, 1121–1130. 21771902 10.1093/schbul/sbr081 PMC3196960

[ref47] Pincus, H. A., Tew, J. D., & First, M. B. (2004). Psychiatric comorbidity: Is more less? World Psychiatry, 3, 18–23. 16633444 PMC1414654

[ref48] Plana-Ripoll, O., Pedersen, C. B., Holtz, Y., Benros, M. E., Dalsgaard, S., de Jonge, P., … McGrath, J. J. (2019). Exploring comorbidity within mental disorders among a Danish National Population. JAMA Psychiatry, 259–270. 30649197 10.1001/jamapsychiatry.2018.3658 PMC6439836

[ref49] Rasmussen, A. R., & Parnas, J. (2022). What is obsession? Differentiating obsessive-compulsive disorder and the schizophrenia spectrum. Schizophrenia Research, 243, 1–8. 35219003 10.1016/j.schres.2022.02.014

[ref50] Robins, E., & Guze, S. B. (1970). Establishment of diagnostic validity in psychiatric illness: Its application to schizophrenia. American Journal of Psychiatry, 126, 983–987. 5409569 10.1176/ajp.126.7.983

[ref51] Strålin, P., & Hetta, J. (2019). First episode psychosis and comorbid ADHD, autism and intellectual disability. European Psychiatry, 55, 18–22. 30384107 10.1016/j.eurpsy.2018.09.007

[ref52] Trull, T. J., Scheiderer, E. M., & Tomko, R. L. (2012). Axis II comorbidity. In Widiger, T. A. (Ed.), The Oxford handbook of personality disorders (pp. 219–236). Oxford: Oxford University Press.

[ref53] van Praag, H. M. (1996). Comorbidity (psycho) analysed. British Journal of Psychiatry Supplement, 30, 129–134. 8864159

[ref54] Vella, G., Aragona, M., & Alliani, D. (2000). The complexity of psychiatric comorbidity: A conceptual and methodological discussion. Psychopathology, 33, 25–30. 10601824 10.1159/000029115

[ref55] Wakefield, J. C. (2016). Diagnostic issues and controversies in DSM-5: Return of the false positives problem. Annual Review of Clinical Psychology, 12, 105–132. 10.1146/annurev-clinpsy-032814-112800 26772207

[ref56] Widiger, T. A., & Ford-Black, M. M. (1994). Diagnoses and disorders. Clinical Psychology: Science and Practice, 1, 84–87.

[ref57] Wittchen, H. U. (1996). What is comorbidity – fact or artefact? British Journal of Psychiatry Supplement, 30, 7–8. 8864143

[ref58] World Health Organization (1992). The ICD-10 classification of mental and behavioural disorders: Clinical descriptions and diagnostic guidelines. Geneva: World Health Organization.

[ref59] Wyrsch, J. (1930). Psychiatrie als Wissenschaft. Neue Schweizer Rundschau, 4, 284–295.

